# Tumor microenvironment-induced tumor cell plasticity: relationship with hypoxic stress and impact on tumor resistance

**DOI:** 10.3389/fonc.2023.1222575

**Published:** 2023-10-11

**Authors:** RF. Zaarour, M. Ribeiro, B. Azzarone, S. Kapoor, S. Chouaib

**Affiliations:** ^1^ Thumbay Research Institute for Precision Medicine, Gulf Medical University, Ajman, United Arab Emirates; ^2^ Tumor Immunology Unit, Bambino Gesù Children’s Hospital, IRCCS, Rome, Italy; ^3^ INSERM UMR 1186, Integrative Tumor Immunology and Immunotherapy, Gustave Roussy, Faculty of Medicine, University Paris-Saclay, Villejuif, France

**Keywords:** cancer stem cell (CSC), hypoxia, hypoxia-inducible factor (HIF), resistance, stemness, tumor microenvironment (TME)

## Abstract

The role of tumor interaction with stromal components during carcinogenesis is crucial for the design of efficient cancer treatment approaches. It is widely admitted that tumor hypoxic stress is associated with tumor aggressiveness and thus impacts susceptibility and resistance to different types of treatments. Notable biological processes that hypoxia functions in include its regulation of tumor heterogeneity and plasticity. While hypoxia has been reported as a major player in tumor survival and dissemination regulation, the significance of hypoxia inducible factors in cancer stem cell development remains poorly understood. Several reports indicate that the emergence of cancer stem cells in addition to their phenotype and function within a hypoxic tumor microenvironment impacts cancer progression. In this respect, evidence showed that cancer stem cells are key elements of intratumoral heterogeneity and more importantly are responsible for tumor relapse and escape to treatments. This paper briefly reviews our current knowledge of the interaction between tumor hypoxic stress and its role in stemness acquisition and maintenance. Our review extensively covers the influence of hypoxia on the formation and maintenance of cancer stem cells and discusses the potential of targeting hypoxia-induced alterations in the expression and function of the so far known stem cell markers in cancer therapy approaches. We believe that a better and integrated understanding of the effect of hypoxia on stemness during carcinogenesis might lead to new strategies for exploiting hypoxia-associated pathways and their targeting in the clinical setting in order to overcome resistance mechanisms. More importantly, at the present time, efforts are oriented towards the design of innovative therapeutical approaches that specifically target cancer stem cells.

## 1 Introduction

Cancer remains one of the leading causes of death worldwide. The mortality rate associated with cancer is high because subpopulations of cancer cells exhibit metastasis. Indeed, metastatic invasion involves complex overlapping processes in which cells undergo multiple steps of reprogramming to promote mechanisms of repair, resistance to cell death, adaptation to changes in metabolism, acquisition of stem cell-like properties, and ultimately, survival. In addition to tumor plasticity and heterogeneity, these subpopulations are further equipped with the capacity to resist therapeutic strategies (1). Tumor heterogeneity refers to the variations observed among tumors of the same type in different patients. This diversity serves as the foundation for the development of personalized treatment approaches aimed at maximizing effectiveness. Furthermore, within a tumor there exists a cellular heterogeneity due to the variable microenvironment shaping the tumor. Hypoxia within solid tumors contributes to this heterogeneity and shapes the behavior of a cell population. As a result, different subpopulations of cancer cells may have differential responses to the same therapy; therefore, the more heterogenous the tumor is, the more likely it will resist therapy and be defined as a treatment-resistant tumor. Within the tumor microenvironment, cancer stem cells (CSCs) are supported by hypoxia and are known to be resistant to therapy. These cells are key to cancer progression and cancer recurrence.

In this review, we examine the existing understanding of the effects of hypoxic stress within the tumor microenvironment (TME) on tumor heterogeneity, plasticity, and resistance. We address the mechanisms that lead to the generation of CSCs, focusing on the potential role of hypoxia in stemness acquisition and maintenance. Identifying CSCs populations within a tumor necessitates a clear understanding of their molecular characteristics, as such we have compiled a set of CSCs markers that are currently recognized as an identifying factor and hence a possible target for therapy. We also discuss the resistant mechanisms that these cells adapt in response to therapy and accordingly, the putative therapeutic strategies for targeting these multifaceted interactions of CSC with TME components.

## 2 Hypoxia, a key factor regulating the tumor microenvironment

The tumor microenvironment (TME) comprises tumor cells, immune cells, signaling molecules, blood vessels and the extracellular matrix (ECM) components (2). An important feature of the TME of solid tumors is hypoxia that arises when oxygen decreases below the level required to maintain tissue homeostasis. The oxygen percentage varies depending on tumor type and has been reported to be as low as 0.3% in pancreatic cancer (6.8% normal tissue), and 1.9% in lung cancer (5.6% normal tissue) (3). Hypoxia ensues due to a decrease in blood oxygen content, or to the non-availability or improper structure of the blood vessels, that could result from increasing proliferative rate of cancer cells (4, 5). The plastic nature of the TME, therefore, results in variations in the severity (mild or severe) and duration of hypoxia (chronic or intermittent).

HIF transcription factors are the main sensors for hypoxia. HIF are comprised of HIF1, HIF2 and HIF3 and include three oxygen sensitive alpha (α) subunits and three nuclear beta (β) subunits. HIFs form a heterodimer of the two subunits α and β. Under normal oxygen tension, the HIF-α subunit is inhibited by Factor Inhibiting HIF (FIH). FIH is an asparagine hydroxylase that hydroxylates HIF and blocks its association with transcriptional co-activators, thus inhibiting its transcriptional activity (6–8). HIF is also degraded following hydroxylation by prolyl hydroxylase (PHD). However, when exposed to low oxygen concentrations, PHD and FIH are down-regulated resulting in the stabilization of HIF-α. HIF-α subsequently translocates to the nucleus, dimerizes with the β subunit and activates the expression of genes that promote carcinogenesis.

HIF-1α is ubiquitously expressed and is degraded under normal oxygen tension, whereas HIF-1β is a stable constitutively expressed nuclear protein. Both proteins have similar DNA sequence specificity, but they differ in their transactivation domains, suggesting that each subunit has distinct roles. This distinct role for HIF-1α and HIF-1β was demonstrated through deletion experiments conducted in mice that showed that HIF-1α and HIF-1β signaling in breast tumors control tumor dissemination in a site-specific manner (9). HIF-1α binds to specific hypoxia responsive elements (HRE) on target genes, it induces NFκB activation resulting in the expression of targets including MIP-2/CXCL2/3, CXCL1 and TNFα (10). Together these induce proliferation of pre-cancerous lesions, thereby facilitating tumorigenesis (11), including invasion of tissues, cell survival, recurrence of tumors, and the formation of CSCs (12, 13).

HIF-2α is highly homologous to HIF-1α and is regulated in a similar fashion through ubiquitin mediated proteasomal degradation, however the expression pattern of the two proteins is distinct: HIF-2α being expressed mainly in vascularized cells, several evidence points to its role in mediating the remodeling and recruitment of vasculature (14). HIF-2α is also found to mediate the chronic hypoxic response (14). In addition to its roles in the induction of EMT (15), and CSCs induction (16), HIF-2α is shown to be essential for T-regs development (17). Furthermore, studies showed that a crosstalk between the expression of HIF-1α and HIF-2α in T-regs contributes to a tumor-suppressive activity (17).

HIF-3α is less studied, and it has been shown to play a role in cancer cell invasion and migration (18). And some research has demonstrated a positive role for HIF-3α in non-small cell lung cancer (NSCLC) (19). However recent work evaluated the expression levels of HIF-3α in various types of cancer, and interestingly found that in contrast to HIF-1α and HIF-2α, an increase in expression levels of HIF-3α correlated with better survival (20). Additional studies are needed to further dissect the role of this protein and its interplay with HIF1 and HIF2 in tumorigenesis of specific cancer types.

Moreover, the hypoxic TME is acidic because HIF1 regulates tumor cells’ metabolic activities, nutrient sensing, and availability (21). In tumor cells glycolysis results from the anaerobic breakdown of glucose due to the lack of oxygen or from aerobic glycolysis (Warburg effect), which further leads to the production of lactate resulting in an acidic microenvironment (21). HIF1 directly plays a role in this process by increasing the expression of pyruvate dehydrogenase kinase, that subsequently inhibits pyruvate dehydrogenase and thereby represses oxygen consumption and redirects pyruvate to be used in glycolysis (21). HIF1 also enhances the expression of glucose transporters and glycolytic enzymes (22). The resultant acidic TME has significant effects on several cells including suppressing the immune response (23).

Finally, the variability in hypoxia directly impacts the behaviors of the tumor vis a vis resistance to therapy and immune escape and renders targeted treatment more challenging (3, 24). HIF protein response to different types of hypoxias plays a role in this by increasing the complexity of the TME. For example, HIF-1α expression is an acute response and it gets degraded in chronic hypoxia, whereas HIF-2α protein levels increase for longer duration. HIF-2α on the other hand is more sensitive to mild hypoxia (5%) compared to HIF-1α (25). In addition, the cyclic nature of hypoxia differentially controls HIFs, HIF-1α increases in cyclic hypoxia but HIF-2α decreases (26) but it is important to note that it is very difficult to monitor and follow the spaciotemporal hypoxia fluctuations in individual tumors. And it is evident that better understanding of the hypoxia response in individual patients would be necessary to initiate effective treatment strategies.

## 3 Hypoxia’s role in the induction and maintenance of cancer stem cells

To adapt to hypoxic stress, cells activate several genes that regulate many pathways (27). Hypoxia plays an important role in the induction of epithelial to mesenchymal transition (EMT), where epithelial cells acquire the capacity to migrate and invade neighboring tissues (28). Hypoxia within the TME plays an important role in CSCs initiation and maintenance (29). CSCs drive tumor initiation, recurrence, metastatic potential, residual disease, and therapy resistance. Furthermore, these cells maintain properties of normal stem cells and are unique in that they are capable of self-renewal and remaining undifferentiated (30). Understanding the processes that give rise to and sustain CSCs is therefore crucial as they are present across various cancer types and targeting them could be detrimental to tumor survival.

While cancer occurs as a sporadic event, resultant from environmental factors (carcinogens, chemicals, biological agents, radiation) or as an inherited event, CSCs origins are debated, and evidence supports that they could originate from existing stem cells or develop following tumor formation. Because cancer can develop from the gradual accumulation of mutations in a single specific cell over a prolonged period, stem cells – that could have long lifespans – have the potential to accumulate mutations that initiate cancer. This is supported by early work where pathologists determined that cancer tissues contain cells that exhibit properties of early embryonic dormant tissue (31) that lead to the “embryonic rest” hypothesis of cancer development which suggested that cancer may arise from embryonic dormant cells that persist in developing organs after embryogenesis (32).

**Figure 1:**
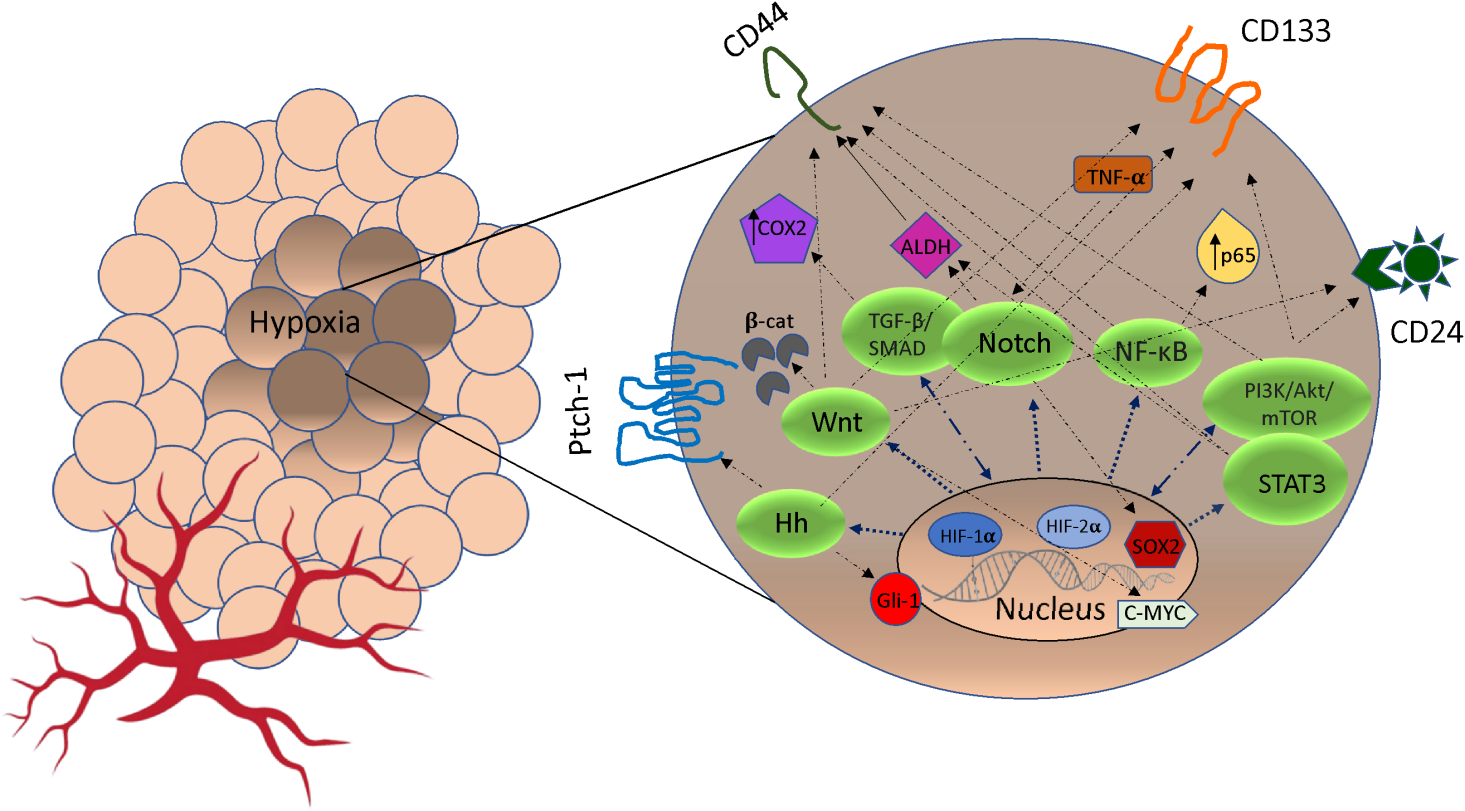
The crosstalk between pathways signaling and markers of cancer stem cells (CSCs) stimulated by hypoxia. Under hypoxic condition, Notch, Wnt, Hedgehog (Hh), NF-κB, TGF-β/SMAD, PI3K/AkT/mTOR and STAT3 pathways can be activated by HIF expression. In some cases, cross-talk between pathways may promote markers expression (CD44, CD24, ALDH1, Patch-1, PROM1, Gli-1, SOX2, c-MYC, p65, TNF-α/β, COX2) that promote phenotypes of CSCs and resistance to cancer therapeutics (35–38). Hh, Hedgehog; TGF-β, Transforming Growth Factor Beta; NF-κB, Nuclear factor-κB; PI3K, intracellular phosphatidylinositol kinase; Akt, serine/threonine kinase; mTOR, mammalian target of rapamycin; STAT3, signal transducer and activator of transcription 3; β-cat, β-catenin; COX2, cytochrome c oxidase subunit 2; ADLH, aldehyde dehydrogenase; TNF-α, Tumor Necrosis Factor Alpha; CD133, Cluster of Differentiation 133; CD24, Cluster of Differentiation 24; CD44, Cluster of Differentiation 44.

In the TME, hypoxia plays a key role in the acquisition of CSCs and this occurs on several fronts. Under hypoxic conditions, signaling pathways regulate stemness, pluripotency and viability of CSC populations. For example, HIF-1α regulates stem cell phenotypes through activation of signaling pathways including Notch, TGF-/SMAD, NF-B, PI3K/Akt/mTOR, STAT3, MAPK/ERK as well as via transcription factors including C/EBPδ, SOX2 and c-MYC (33, 34) (**Figure 1**). In hypoxic areas, HIF-1α protein has been shown to activate Notch, Wnt, and Hedgehog (Hh) pathways target genes, inhibiting their differentiation and stimulating stem cell self-renewal and multi-potency (39). The Notch receptors, along with the Jagged or Delta ligand family, translocate into the nucleus to create a DNA-binding complex. Cofactors like mastermind-like (MAML), CSL, NICD, and p300, together promote the activation of Notch target genes. This process significantly contributes to the maintenance of the stem cell population (39, 40). Furthermore, hypoxia and HIF factors have also been shown to enhance the propagation of CSCs identified by the upregulation of CSC markers including CD133, Sox2, Oct4, CD44, and ALDH (34) (**Figure 1**). This suggests a particular role for HIF in cancer cell phenotype and plasticity. Finally, hypoxia through HIF-1α activation induces methylation of genes promoting CSCs. Recent evidence has shown that mutated DNMT3a, in a mechanistic manner, can activate specific enhancers, resulting in localized DNA methylation and histone acetylation changes. These alterations ultimately lead to disruptions in stemness pathways (41). Moreover, epigenetic modifications of the DNA packaging protein Histone H3 (H3Kme3 and H3K27Ac) at the promoter region of IFN-γ increases the expression of IFN stimulated genes, including PD-L1 expression (42). PD-L1 in turn promotes CSC expansion (43).

Another factor in the TME that contributes to tumor progression and sustains CSCs is the intercellular communication between cancer and stromal cells within the TME. Recent studies demonstrated that non-CSCs could become CSC by processes mediated by secreted factors. Indeed, proteins, cytokines, chemokines, microRNAs, and other substances could be secreted by cancer cells to mediate tumor maintenance and CSCs formation (44–46). It has been reported that IL-6 present in the TME can induce non-CSCs to transform into tumor stem cells (47, 48). Likewise, tumor associated mesenchymal stem cells through direct contact with cancer cells have been shown to promote CSCs features through pathways involving microRNAs (49). Cancer-associated fibroblasts (CAFs) release hepatocyte growth factors (HGF) and annexin A1 which have the ability to revert differentiated tumor cells back to stem cell-like phenotypes (50). An additional example that highlighted the importance of intercellular communications in tumorigenesis came from work that demonstrated that macrophages, could secrete an increased amount of the cytokine osteopontin (OPN) when cocultured with CD44-positive cancer cells, that subsequently promoted tumorigenicity (51). Macrophages secrete oncostatin-M, an IL-6 family cytokine, that can activate the dedifferentiation of non-CSCs into aggressive CSCs (52). Cancer associated fibroblasts can also modulate CSC plasticity through signaling pathways including IGF-II/IGF1R; FAK and c-Met/FRA1/HEY1 (53–55). The activation of NOTCH1 signaling by dermal fibroblasts derived from mesenchymal stem cells was observed to regulate both plasticity and stemness (56). Therefore, the interaction of the microenvironment facilitates the plasticity and stemness of these cells and can be modulated by the secretome.

## 4 Cancer stem cells markers and hypoxia inducible factors

Several specific markers are established to categorize cancer stem cells, enabling their identification, purification, and potential use for targeted therapies (**Table 1**). These markers’ expression is regulated by spatial and temporal characteristics, indicating the remarkable adaptability of these cells (152). In 1997, the first evidence of the existence of CSCs surfaced from experiments that demonstrated the existence of a subpopulation of CD34-expressing cells in leukemia. These cells were capable of initiating tumors in NOD/SCID mice that resembled the donor’s tumor (32). Following this, in 2002 it was shown that cancer stem like sphere-forming cells from human gliomas could induce tumors resembling the original tumor when transplanted intracranially in nude mice (153, 154). As the research progressed, additional specific markers of CSCs were identified. The most common markers that are in use for CSC isolation are CD133 (also known as PROM1), CD44, ALDH1A1, CD34, CD24 (155, 156). Indeed, CD133 positive cells isolated from colon carcinomas could grow as tumor spheroids *in vitro* as well as initiate tumor growth when xenografted in immunodeficient mice (157). Hypoxia and HIF transcription factors play a key role in the expression of these markers, however the molecular mechanisms leading to the increased expression is not yet understood for several of these markers (**Table 1**). It should be noted that due to the expression of a heterogeneous range of stem cell markers, cancer stem cells that originate from a hypoxic microenvironment may exist in a distinct stem cell states or exhibit variations in the expression of different sets of stem cell markers. Targeting CSCs markers could be effective in treatment strategies. Indeed, downregulation of CD133 using short hairpin RNAs led to slower cell growth of human metastatic melanoma and slowed the spheroid formation and metastatic potential of melanoma (158).

**Table 1:** Relationship of various cancer stem cell markers with hypoxia in selected solid tumors (Lung, Breast, Pancreatic, Gastric, Prostate, Bladder).

CSC marker/pathway	Type of cancer	Hypoxia relationship	Reference
Membrane			
**CD24**	Lung, Breast, Pancreatic, Gastric, Prostate, Bladder	The expression of CD24 is induced by HIF1 through binding to HRE element in the CD24 promoter (57)	(58–61)
**CD44**	Lung, Breast, Pancreatic, Gastric, Prostate	HIF-2α binds to CD44 enhancing HIF target gene activation (62)	(59–61, 63)
**CD47**	Lung, Breast, Pancreatic, Gastric, Prostate	HIF-1 directly binds to HRE elements in CD47 (64)	(65–69)
**ITGA6/CD49f**	Breast, Gastric, Colon, Prostate	HRE elements in the ITGA6 promoter are specific for HIF-1α or HIF-2α (70).	(71)
**ICAM1/CD54**	Breast, Gastric, Prostate	Hypoxia upregulates CD54 (72)	(59, 73, 74)
**PLAUR/CD87**	Lung, Breast, Pancreatic	Hypoxia increases the expression of PLAUR (75)	(58, 76)
**THY1/CD90**	Lung, Breast, Pancreatic, gastric, prostate	HIF-1 target genes (cytoines, and growth factors) increase the expression of Thy-1 (77)	(58, 78–81)
**SLC3A2/CD98**	Lung, Pancreatic, Gastric, Prostate	Hypoxia upregulates CD98 that promotes tumorigenesis (82)	(83, 84)
**KIT/CD117**	Lung, Breast, Pancreatic, Gastric, Prostate	Activated c-KIT enhances nuclear HIF-1α levels (85)	(58, 86–89)
**PROM1/CD133**	Lung, Breast, Pancreatic, Gastric, Prostate	Hypoxia can upregulate or downregulate CD133 (90, 91)	(58, 92–94)
**ALCAM/CD166**	Lung, Breast, Pancreatic, Gastric, Prostate	CD166-positive stem cells acquire CSCs features and drug resistance in response to chemical induced hypoxia (95)	(58, 96–99)
**EpCAM/ESA**	Lung, Breast, Pancreatic, Gastric	Hypoxia influences stem cell characteristics and triggers EMT via N-glycosylation of EpCAM (100)	(101)
**ABCB1**	Lung, Breast, Pancreatic, Gastric, Prostate	HIF-1α dependent regulation (102)	(61, 103, 104)
**ABCG2**	Lung, Breast, Pancreatic, Gastric, Prostate	Hif-1 results in the increased expression of ABCG2,; HIF-2α positively correlated with ABCG2 expression (105)	(58, 105–108)
**FZD**	Lung, Breast, Pancreatic, Gastric, Prostate	HIF1is required for the expression of FZD (109)	(109–111)
**CXCR4**	Lung, Breast, Pancreatic, Gastric, Prostate	Hypoxia stabilizes HIF-1α to upregulate CXCR4 (112)	(58, 61, 113, 114)
**PODXL1**	Lung, Breast, Pancreatic, Gastric	Not determined	(58, 115, 116)
**LOX**	Lung, Breast, Pancreatic, Gastric	HRE elements in LOX promoter specific to HIF-2α (117)	(118–120)
**TIE1**	Lung, Pancreatic, Gastric	Tie1 expression is mediated by HIF-1α binding to HRE elements in the Tie1 promoter (121)	(122–124)
Intracellular			
**ALDH1A1**	Lung, Breast, Pancreatic, Gastric, Prostate, Bladder	Hypoxia upregulates ALDH1 expression (125)	(126)
**SOX2**	Lung, Breast, Pancreatic, Gastric, Prostate, Bladder	HIF-dependent demethylation of SOX2 mRNA leading to increased expression (127)	(128)
**NANOG**	Lung, Breast, Pancreatic, Gastric, Prostate	HIF1 recruits NANOG to activate transcription TERT. TERT expression is required for stem cell self-renewal (129)	(130)
**POU5F1/OCT4**	Lung, Breast, Pancreatic, Gastric	HIF-2α, induces the expression of Oct-4 promoter and induces its expression (131)	(132)
**BMI1**	Lung, Breast, Pancreatic, Gastric, Prostate, Bladder	Hypoxia-induced increase BMI-1 (133)	(134)
**DCLK1**	Lung, Breast, Pancreatic, Gastric, Prostate, Bladder	Under hypoxia, HIF-1α activates KDM3A52, which in turn, increased DCLK1 mRNA expression (135)	(61, 136–141)
**PKM2**	Lung, Breast Pancreatic, Gastric, Prostate, Bladder	HIF-1α induces the expression of PKM2 expression in induced by HIF-1α through binding HRE elements in the PKM2 promoter (142)	(143–148)
**KLF4**	Lung, Breast, Pancreatic, Gastric, Prostate	KLF4 expression is induced by HIF-1α through binding HRE elements in the KLF4 promoter (149)	(150, 151)

CD24, Cluster of Differentiation 24; CD44, Cluster of Differentiation 44; CD47, Cluster of Differentiation 47; ITGA6, Integrin Subunit Alpha 6; ICAM1, Intercellular Adhesion Molecule 1; PLAUR, Plasminogen Activator, Urokinase Receptor; THY1, Thymocyte Nuclear Protein 1; SLC3A2, Solute Carrier Family 3 Member 2; KIT, Receptor Tyrosine Kinase; PROM1, Prominin 1; ALCAM, Activated Leukocyte Cell Adhesion Molecule; EpCAM, Epithelial Cell Adhesion Molecule; ABCB1, ATP-Binding Cassette Transporter 1; ABCG2, ATP-Binding Cassette Gene 2; FZD, Frizzled Class Receptor; CXCR4, C-X-C Motif Chemokine Receptor 4; PODXL1, Podocalyxin-Like Protein 1; LOX, Lysyl Oxidase; ALDH, Aldehyde Dehydrogenase; SOX2, Sex-Determining Region Y-box 2; OCT4, Octamer-Binding Transcription Factor 4; POU5F1, POU class 5 homeobox 1 BMI1, B-Cell-Specific Moloney Murine Leukemia Virus Integration Site 1; DCLK1, Doublecortin Like Kinase 1; PKM2, Pyruvate Kinase; KLF4, Krüppel-Like Factor 4; Tie1, Tyrosine Kinase With Immunoglobulin Like And EGF Like Domains 1.

## 5 Mesenchymal stem cells within the tumor microenvironment contribute to resistance mechanisms

Hypoxia also influences mesenchymal stem cells (MSCs) features including differentiation cell viability, proliferation capacity, migration, and metabolism. It has been reported that the intratumoral MSC (T-MSC) play a key role in tumor progression and immune regulation (159, 160). T-MSC are either Bone Marrow-derived MSC that migrate and infiltrate the TME (161, 162). Alternatively, as demonstrated in the Wilm’s tumor, T-MSC represent the neoplastic mesenchymal tissue that is originated by a common neoplastic stem cell that also generates the blastemal and the epithelial components (163). T-MSC may be also directly generated by particular tumor cell subsets as reported in the neuroblastoma (164) Immunohistochemical analysis and *in vitro* studies show that T-MSC establish direct cellular crosstalk with the tumor cells (159), and/or pro inflammatory cells such M2 macrophages (165, 166). In addition, several tumor-associated inflammatory markers such as COX-2, nitric oxide synthase and nitrotyrosine, may be detected within the tumor stroma (159, 161, 165). In addition T-MSC (tumor-associated mesenchymal stem cells), possess potent immunosuppressive properties that can affect T cells (161), impair the cytolytic functions of NK cells and induce the polarization of monocytes towards alternatively activated macrophages (M2) (164, 166–169) this may, in turn, further compromise the functions of NK cell (168). Efficient tumor elimination requires a combined action both on tumor cells and stromal components (159, 170). As future perspective, to eliminate or disable T-MSC *in vivo* could involve targeting of the mesenchymal marker TRC105 with the TRC105 monoclonal antibodies (mAb) exploiting their ability to induce antibody-dependent cell mediated cytotoxicity (ADCC) (167, 170), or inducing their senescence through the application of anti-cancer drugs such as isoalantolactone which has demonstrated effectiveness both *in vitro* and *in vivo.*

## 6 Mechanisms of CSCs resistance to therapy

Hypoxia in the TME induces and maintains CSCs which are equipped with mechanisms to evade treatment modalities. In addition, hypoxia confers radiotherapy resistance through an increase in reactive oxygen species (ROS). Although ROS, induced by radiation therapy or hypoxia, triggers DNA damage and cell death, this effect is mitigated under hypoxic stress by the induction of antioxidant HIF target genes resulting in ROS buffering action (171).

CSCs populations resist therapy and could also increase in response to therapy. Because CSCs are predominantly in the G0 or resting phase of the cell cycle, they escape conventional treatment regimens focused on eradicating proliferating tumor masses (172). Unlike differentiated cells that undergo apoptosis, non-cancerous stem cells do not, and this is important because it enables them to restore and rebuild normal organs following damage. CSCs like normal stem cells have mechanisms of resistance to apoptosis. Indeed, CSCs express high levels of antiapoptotic proteins such as Bcl-2 family proteins and inhibitors of apoptosis (173–176). Overexpression of Bcl-2 protein in the hematopoietic system results in an increase in hematopoietic stem cell number and chemoresistance (177, 178). Furthermore, stem cells possess asynchronous DNA synthesis activity and increased DNA repair activity (179). During asynchronous DNA synthesis, the parental ‘immortal’ DNA strand consistently segregates with the stem cell rather than the differentiating progeny. This segregation process may be regulated by P53 (180). As a result, stem cells gain an advantage by avoiding the accumulation of mutations related to replication and the detrimental effects of DNA-damaging agents and antiapoptotic proteins. Moreover, there are populations of stem cell-like cells found in many tumors that have been shown to express high levels of multidrug efflux pumps (MDR) or transporter proteins/detoxification proteins, such as MDR1, ABCB1, ABCG2 (BCRP) that play a significant role in expelling cytotoxic drugs from cells leading to high resistance to chemotherapeutic agents (179). The overexpression of ABC protein is a critical protective mechanism for CSCs in response to chemotherapy (152, 181). Indeed, numerous studies have reported that CSCs exhibit more resistance to chemotherapy and/or radiotherapy compared to differentiated tumor cells (182–184) in various types of cancer including breast cancer (185), ovarian cancer (186), colon cancer (157, 187) lung cancer (188, 189) and other deadly forms of cancers such as pancreatic cancers (190), myeloma (191, 192) and leukemia (193). *In vivo* and *in vitro* studies of common cancers have demonstrated resistance of CSCs to standard chemotherapy agents such as: oxaliplatin and 5-fluorouracil in colorectal cancers (194), cisplatin and paclitaxel in ovarian cancers and docetaxel and doxorubicin in breast cancers (195). These studies demonstrated that these cells are less susceptible to chemotherapy when compared to differentiated cells. A study by Chen et al. in 2012 showed that after treatment with temozolamide (TMZ) there was complete restoration of tumor cell population when CSCs were present (196). An additional study conducted in breast cancer revealed that taxane treatment could increase the generation of CSCs and further contribute to therapy resistance (197). Furthermore, after undergoing standard chemotherapy treatment with docetaxel, doxorubicin, cyclophosphamide and trastuzumab, breast cancer cells that are CD44+ and CD24- were found to exhibit resistance to chemotherapy. Specifically, 12 weeks post-chemotherapy the population of CD44+CD24-/low cells, increased from 4.7% to 13.6% while the proportion of epithelial cancer cells remained relatively unchanged (198). These findings suggest that CD44+CD24-/low cells may play a significant role in mediating chemotherapy resistance in breast cancer.

Another important reason for cancers resistance to chemotherapy drugs like cisplatin, etoposide, fluorouracil, and gefitinib is the overexpression of cytosolic enzyme called aldehyde dehydrogenase (ALDH) that protects cells from the toxic effects of elevated levels of reactive oxygen species (ROS) (199, 200). ALDH proteins scavenge free radicals generated by oxidative stress induced by radiation or drugs (201). However, when the activity of ALDH is inhibited in therapy-resistant CSCs, it results in the accumulation of excessive ROS. This accumulation of ROS leads to DNA damage and triggers apoptosis causing toxic effects on CSCs (199). patients with resectable esophageal cancer who exhibit high expression levels of ALDH1 are predicted to experience a poor response or resistance to preoperative chemotherapy (202, 203). Moreover, the CSC population can present clonal variation as well as distinct CSC-driven clones that differ in their growth rate or resistance to therapy, modeling tumor behavior. Finally, CSCs of various cancers not only develop chemoresistance, but they also develop resistance to radiation therapy leading to failure of treatments (204). Therefore, targeting CSCs utilizing their unique cell surface markers to develop antibodies or antibodies-drug conjugates could be more effective (**Tables 1**–**3**) (158).

**Table 2:** Examples of targeting approaches for cancer stem cells.

Targeting Approach	Mechanism of Action	Examples
Differentiation	Induce CSCs to differentiate into non-tumorigenic cells	ATRA in acute promyelocytic leukemia (APL). BMP pathway activators (205, 206).
Inhibition of Self-renewal	Block CSCs’ ability to self-renew and proliferate	Notch pathway inhibitors (e.g., DAPT, RO4929097). Wnt signaling pathway inhibitors (e.g., LGK974, ICG-001) (207)
Targeting Surface Markers	Specific antibodies or ligands targeting CSC-specific surface markers	CD44-targeting antibodies in breast cancer. CD133-targeting agents in brain tumors (208, 209)
Metabolic Targeting	Exploiting distinct metabolic pathways in CSCs	Metformin targeting CSCs in breast cancer. Salinomycin targeting CSCs in colorectal cancer (210, 211)
Targeting/Modulation of Signaling Pathways	Interfering with crucial signaling pathways driving CSCs Inhibits critical signaling pathways that maintain CSC self-renewal and survival.	Hedgehog pathway inhibitors (e.g., Vismodegib in basal cell carcinoma) STAT3 inhibitors (e.g., Napabucasin in pancreatic cancer). Notch signalling pathway inhibitors in breast cancer. Hedgehog pathway inhibitors in medulloblastoma (207, 212–214).
Combination Therapy	Simultaneously targeting CSCs and bulk tumor cells	Combination of CD47-blocking antibodies with chemotherapy. Combination of CSC-targeting agents with radiotherapy (215, 216)
Immunotherapy	Enhances the immune system’s ability to recognize and attack CSCs by targeting specific antigens on their surface.	Chimeric Antigen Receptor (CAR) T-cell therapy targeting CD19 in leukemia. Dendritic cell vaccines targeting CSC-specific antigens (217, 218)
Drug Resistance Inhibition	Targets mechanisms that confer drug resistance to CSCs, making them more susceptible to standard therapies.	ABC transporters inhibitors like Verapamil to overcome CSCs’ efflux pump-mediated resistance, BCL-2 inhibitors in leukemia to counter apoptosis resistance (219).
Nanoparticle-based Delivery	Utilizes nanoparticles to deliver therapeutic agents specifically to CSCs, increasing treatment efficacy and reducing systemic toxicity.	Targeted liposomal delivery of siRNA against CSC-associated genes. Encapsulation of chemotherapeutic drugs in nanoparticles for CSC-targeted therapy (220).
Epigenetic Modulation	Alters the epigenetic landscape of CSCs, affecting their gene expression patterns and cellular functions.	DNA methyltransferase inhibitors (e.g., Decitabine) in leukemia, HDAC inhibitors in solid tumors (221, 222)

ATRA, all-trans retinoic acid; BMP, bone morphogenetic protein; HDAC, Histone deacetylase.

**Table 3:** Current therapies targeting cancer stem cell pathways.

Therapy	Targeted Pathway(s)	Cancer Type(s)	Publication/Trial
Notch inhibitors	Notch signaling pathway	Various solid tumors	(223–225)
Hedgehog inhibitors	Hedgehog signaling pathway	Basal cell carcinoma, medulloblastoma	(223, 226–229)
VEGF inhibitors	VEGF studies		(230–232)
ALDH inhibitors	ALDH enzyme	Breast cancer, lung cancer, etc.	(223, 233)
CD44-targeted therapy	CD44 protein	Breast cancer, pancreatic cancer	(223, 234)
BMI1 inhibitors	BMI1 gene/protein	Various solid tumors, leukemias	(223, 235)
STAT3 inhibitors	STAT3 signalling pathway	Glioblastoma, breast cancer, colorectal cancer	(223, 236–239)
Wnt pathway inhibitors	Wnt signalling pathway	Colorectal cancer	(240–247)
CXCR1/2 inhibitors	CXCR1 and CXCR2 receptors	Colorectal cancer, pancreatic cancer	(248, 249)

VEGF, vascular endothelial growth factor; ALDH, Aldehyde Dehydrogenase; CD44, Cluster of Differentiation 44; BMI1, B-Cell-Specific Moloney Murine Leukemia Virus Integration Site 1; STAT3, signal transducer and activator of transcription-3; Wnt, windless/integrated; CXCR1/2 C-X-C chemokine receptor type ½.

## 7 Hypoxia and autophagy in the regulation of cancer stem cells

Autophagy is a self-eating mechanism that is activated in response to stress in order to sustain homeostasis and cell survival. Many studies addressed the role for autophagy in self-renewal, pluripotency, and differentiation of normal stem cells (250, 251). In the context of cancer autophagy could function to either prevent or promote cancer, depending on the cancer stage (252). Furthermore, CSCs within a tumor are characterized by a high level of autophagy. Autophagy is upregulated in mammospheres (representing stem-like cells) compared to parental adherent cells (253). In addition to breast CSCs (253, 254), autophagy has been linked to CSCs in liver (255), pancreatic (256), osteosarcoma (257), ovarian (258) and glioblastoma (259).

**Figure 2:**
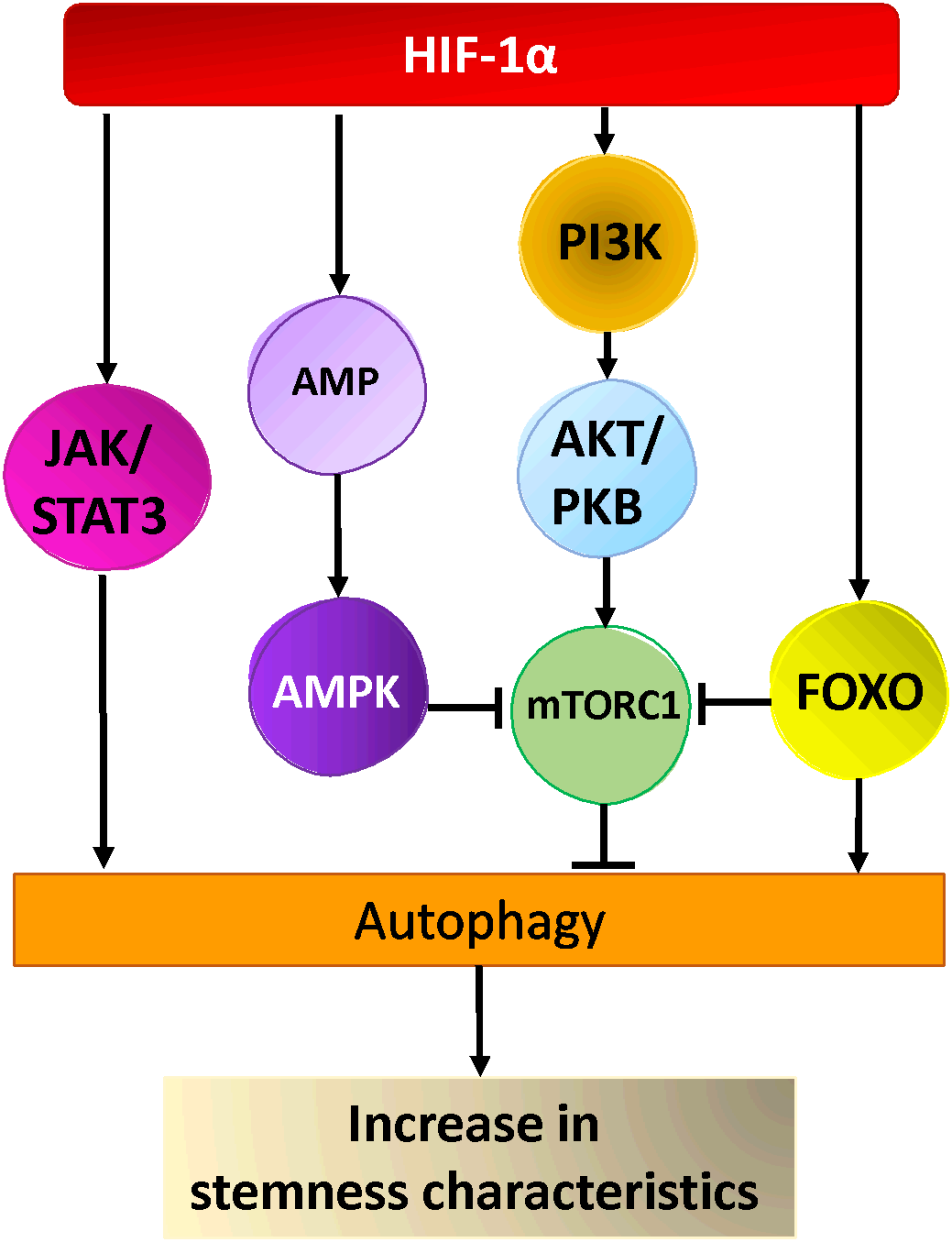
Hypoxia activated pathways in cancer stem cells. HIF-1α activates AKT via PI3-Kinase, leading to the activation of mTOR-C1, AMPK and FOXO activation by HIF-1α leads to inhibition of mTORC1 and autophagy activation. Hypoxia activates the JAK/STAT pathway via HIF-1α resulting in the activation of autophagy and cancer stem cells maintenance/generation.

Ongoing research is focused on elucidating the mechanisms through which autophagy contributes to the maintenance of stemness, as well as understanding the reliance of stem cells on autophagy (260). Numerous studies have shown that autophagy regulates the maintenance of pluripotency and homeostasis of CSCs under various pathophysiological conditions (260–262). Two important autophagy proteins, Beclin1 and Atg4a, were found to be critical for the maintenance and expansion of breast CSCs as well as tumor development in nude mice (253, 254). In the same line of thought, the suppression of autophagy by knocking down ATG5 and ATG7 drastically decreases the stemness characteristics of colorectal CSCs. This is evident through the decrease of stemness markers such as OCT4, SOX2, and NANOG, the induction of cellular senescence, and the decline of the proliferative capacities of CSCs in tumors (263). In another study, it was shown that inhibition of autophagy by knockdown of ATG7 or BECN1 modified the CD44+/CD24low/- (stem cell phenotype) population of breast cancer cells by regulating CD24 and IL-6 secretion (264). Indeed, IL-6 secretion was crucial for CSC maintenance, mammosphere formation, and conversion of non-CSCs into CSCs in different breast cell lines and a prostate cell line (264, 265). Moreover, autophagy inhibition decreased the secretion of IL-6 (264), most likely via JAK2/STAT3 signaling pathway, which was preferentially active in breast cancer cells compared with other tumor cell types (266). It is worth noting that, STAT3 has been reported to regulate the expression of multiple autophagy genes, including ATG3, BECN1, and BNIP3 (267). In a study involving a mouse model of breast CSCs two distinct signaling pathways were identified (268) (**Figure 2**). Yeo and colleagues isolated two subpopulations of breast CSCs, one luminal one (ALDH+) and one mesenchymal one (CD29high/CD61+). Intriguingly, stemness markers (ALDH, CD29, CD61) were downregulated in both populations following depletion of FIP200 (a component of ULK1 complex), which was correlated with decreased EGFR/STAT3 and TGF beta/SMAD signaling (268). Taken together, these findings indicate that the activation of STAT3 signaling could play an important role in the development of CSCs.

Other research suggested that the fate of various CSCs could also be regulated by FoxO3a protein, a member of the Forkhead box O protein family. Indeed, the modulation of FoxO3a in breast cancer, affected CSC markers expression and had an impact on the formation of mammospheres as well as breast cancer-initiating potential (269). Moreover, the knockdown of FoxO3a led to an increase in the self-renewal capacity of the prostate, colorectal, and ovarian cancer stem cells as well as their tumorigenic potential (270–272). FoxO3a, which integrates the signals from Akt and Erk pathways, also plays a pivotal role in the control of differentiation and tumorigenicity of glioma CSCs (273). Several studies showed that FoxO transcription factors (including FoxO3a) are able to induce the expression of multiple ATG genes (267). Additionally, cytosolic FoxOs are able to regulate autophagy by interacting directly with cytosolic autophagy protein (267)(**Figure 2**). Nevertheless, further investigation is necessary to understand how FoxO-dependent autophagy and FoxO-dependent regulation of stemness are interrelated in tumorigenesis. Recently, a new link between autophagy and stemness was discovered, showing that Forkhead box A2 (FOXA2) is highly expressed in ovarian CSCs and modulates autophagy. Inhibition of autophagy induces FOXA2 downregulation and impairment of the self-renewal ability of ovarian CSCs.

In glioblastoma, several regulators of autophagy are highly expressed in tumors with a mesenchymal signature. Notably, the key regulator of selective autophagy p62/SQSTM1 and DNA damage-regulated autophagy modulator 1 (DRAM1) are both highly expressed in glioma CSCs (GSCs). Knockdown of DRAM1 and p62/SQSTM1 in GSCs leads to alteration of cellular bioenergetics and inhibits their migratory and invasive abilities. Moreover, these data suggest that the RAS/MAPK pathway may positively modulate autophagy in GSCs (274). However, other studies indicate that autophagy can regulate the differentiation of GSCs. The enhancement of autophagy promotes differentiation, whereas inhibition of autophagy suppresses differentiation (275, 276). Thus, it is unclear whether autophagy regulates stemness in glioma stem cells and it requires further elucidation.

Interestingly, in hematological malignancies, autophagy’s function could be reversed depending on the type of progenitors and the state of leukemia expansion (tumor initiation vs progression). In chronic myeloid leukemia (CML), inhibition of autophagy by silencing ATG7 or ATG4B curbs the expansion of CML CD34+ stem/progenitor cells (277, 278). Conversely, in acute myeloid leukemia (AML), monoallelic loss of a key autophagy gene *Atg5* is sufficient to accelerate the disease progression and aggressiveness in a mouse AML model (279). Altogether, this evidence highlights that CSCs are often characterized by an increase in autophagy that maintains their pluripotency. However, different signaling pathways could be responsible for autophagy-dependent CSCs maintenance. Probably the mechanisms that underlie these activities depend on the cell type or malignancy degree. Therefore, the governing role of autophagy in CSCs is complex, and additional research is necessary.

## 8 Targeting cancer stem cells is a promising approach to cancer treatment

CSCs are accountable for not only the formation, progression, and spread of tumors but also for resistance to treatment. Therefore, targeting CSCs specifically may be an appropriate approach to combat cancer. Because CSCs are used as a detection index, the availability of assays that allow detection and identification of CSCs after tumor initiation is of high relevance to guide treatment modalities. Furthermore, as chemotherapy or radiotherapy are known to target dividing cells, development of improved screening and targeting strategies could bring new perspectives to cancer exploration and cancer therapy (**Table 2**). While targeting CSCs holds promise, it also presents significant challenges. The development of therapies that selectively target CSCs without affecting normal stem cells remains a huge challenge. The identification and isolation of CSCs can be complex due to their heterogeneity and dynamic nature. However, the pursuit of CSC-targeted therapies in the context of hypoxic cancer environments presents an exciting avenue for advancing cancer treatment strategies and improving patient outcomes. In hypoxic cancer environments, targeting cancer stem cells (CSCs) has gained considerable attention as a promising approach to cancer treatment due to the following facts:

1. CSCs Contribute to Tumor Progression and Recurrence: Cancer stem cells are thought to be responsible for initiating tumors, driving their growth, and contributing to disease recurrence after treatment. They possess self-renewal and differentiation capabilities, allowing them to regenerate the entire tumor hierarchy. Targeting CSCs aims to disrupt this regenerative potential and halt tumor progression.
2. Resistance to Conventional Therapies: CSCs have been shown to exhibit increased resistance to various conventional cancer treatments, such as chemotherapy and radiation therapy. This resistance is due to their slow-cycling nature, enhanced DNA repair mechanisms, and expression of drug efflux transporters. By targeting CSCs, researchers aim to overcome the limitations posed by treatment-resistant cell populations.
3. Tumor Heterogeneity and Plasticity: Hypoxic environments in tumors can promote genetic and phenotypic heterogeneity, contributing to therapy resistance. CSCs are often associated with this heterogeneity and plasticity, making them a key target for therapy to prevent the emergence of treatment-resistant cell populations.
4. Microenvironmental Adaptation: CSCs are known to adapt to the hypoxic tumor microenvironment by upregulating hypoxia-inducible factors (HIFs) and other survival mechanisms. Targeting these adaptations can sensitize CSCs to therapy and disrupt their ability to survive under adverse conditions.
5. Reducing Relapse: Eliminating CSCs can reduce the likelihood of disease relapse. If CSCs are not effectively targeted, they can remain dormant and later give rise to new tumors, contributing to relapse and metastasis.
6. Long-Term Treatment Efficacy: By targeting CSCs, the goal is to achieve long-term treatment efficacy by eradicating the cell population responsible for initiating and sustaining the disease. This approach could lead to more durable responses and improved patient outcomes.
7. Combination Strategies: Targeting CSCs can be combined with conventional therapies to create synergistic effects. By targeting both the bulk of the tumor and the CSC subpopulation, treatment effectiveness may be enhanced.
8. Personalized Medicine: Understanding the molecular characteristics of CSCs and their responses to hypoxia can facilitate the development of personalized treatment strategies. Tailoring treatments to target CSCs based on individual patient profiles could enhance treatment outcomes.
9. Emerging Therapeutic Approaches: Researchers are actively exploring innovative approaches to target CSCs, including the use of specific antibodies, nanoparticles, gene therapies, and small molecules that inhibit key signaling pathways responsible for CSC maintenance.
10. Advancements in Research: As our understanding of CSC biology and the impact of hypoxia on these cells improves, more precise and effective targeting strategies can be developed.

Shown in **Table 2** are examples of approaches developed to target and eradicate cancer stem cells and in **Table 3** are current therapies targeting CSC pathways. By inhibiting signaling pathways specific to cancer stem cells researchers can prevent cancer stem cells from surviving and multiplying (223).

Developing immunotherapeutic approaches to target specific markers on cancer stem cells could be highly effective (280–283), however, it is important to note that as CSCs and normal stem cells share common markers, these types of treatments may result in adverse non desirable effects and therefore would have to be addressed prior to execution. Through differentiation therapy CSCs are induced to differentiate into non-tumorigenic cells. By doing so, researchers can prevent cancer stem cells from proliferating and spreading (284–286). The use of natural compounds such as curcumin, resveratrol, and sulforaphane are being investigated for their potential as CSCs targeting agents because they have been shown to have anti-CSCs properties (287). Finally, nanoparticles can be designed to selectively target CSCs for direct delivery of therapeutic agents resulting in improved efficacy and reduced toxicity (288–290).

## 9 Targeting quiescence as a novel cancer stem cell targeting strategy

Another potential strategy to target CSCs is by inducing them to exit their quiescent state, which is a state of dormancy that allows CSCs to resist chemotherapy and radiation and enter the cell cycle (291). By targeting quiescence, it may be possible to sensitize CSCs to conventional therapies and reduce the risk of tumor recurrence. Several approaches have been proposed to target quiescence in CSCs. One strategy is using drugs to target the signaling pathways that regulate quiescence, such as the Notch, Wnt (223), and Hedgehog pathways. Another approach is to use drugs that interfere with the interactions between CSCs and their microenvironment, which play a critical role in maintaining CSC quiescence (292–294). In addition to drug-based approaches, physical and mechanical cues can also be used to target CSC quiescence. For example, mechanical stress or compression can induce CSCs to exit their quiescent state and become more susceptible to chemotherapy. Another approach is to target the unique metabolic features of quiescent CSCs. CSCs generate energy mainly through glycolysis, (Warburg effect) and this results in rapid ATP production, in the presence of abundant glucose. As quiescent cells they have different metabolic requirements than proliferating cells, which can be exploited for therapy. For example, targeting the metabolism of quiescent CSCs with drugs that inhibit mitochondrial metabolism or fatty acid oxidation has been shown to selectively kill these cells (295). Overall, targeting quiescence represents a promising strategy for destroying CSCs and improving the effectiveness of conventional cancer therapies. However, more research is needed to fully understand the mechanisms underlying CSC quiescence and to develop effective strategies for targeting this state. While there is still much to learn about CSC biology and quiescence, ongoing research in this area holds great promise for the future of cancer treatment.

## 10 Novel therapies for targeting CSCs in the tumor microenvironment and future perspectives

Despite significant efforts and progress made in comprehending cancer over the years, the fact remains that tumors can relapse, metastasize, and recur. While the discovery that tumors possess a heterogeneous population of cells, a subset of which with stem cell-like characteristics, was fascinating and promising, their actual significance in clinical settings was still uncertain. Numerous studies provided increasing evidence for the clinical significance of CSCs and for the important role of hypoxia in supporting stemness. Given CSCs presence in tumors, it is reasonable to assume that targeting them could be the most effective approach to achieve complete cancer elimination. In 2016, Piero and Debashish demonstrated that stem cells make up 4 to 7% of the cells within colorectal tumors, and that patients with this population have a greater likelihood of relapse, even in the second stage of the disease. Furthermore, chemotherapy does not appear to benefit these patients (296). Therefore, developing a more effective method to analyze CSCs within a tumor will be important.

Many pathways that CSCs utilize for their survival have been identified and studied and they present a viable option as cancer therapy targets. Clinical trials are underway to assess the effectiveness of agents targeting stemness pathways, such as Wnt, TGFβ, Notch, Hedgehog, and JAK/STAT, for a broad spectrum of cancers. These agents are being evaluated alone or in combination with conventional therapies, with the goal of completely eradicating the cancer (**Tables 2**, **3**) (297).

## 11 Conclusion

The cellular and metabolic TME is currently attracting a lot of interest given its key role in carcinogenesis. In addition, strong evidence has been provided indicating that tumor heterogeneity represents a serious obstacle for therapy. Carcinogenesis and resistance mechanisms seem to be overlapping, carcinogenesis could endow cells with resistance, but resistance may not dictate carcinogenesis, and to determine conclusively if one mechanism contributes solely to carcinogenesis or resistance exclusively would have to be tested in one context excluding the other, which is not usually tested as most research assumes overlap of the two. Hypoxia clearly can exert several effects on the emergence and function of cancer stem cells, that play roles in both carcinogenesis and resistance. In this respect, the microenvironmental hypoxia is able to induce the alteration of gene expression, and HIF proteins can modulate several CSCs characteristics. It has been reported that microenvironmental hypoxia is able to mediate its effects by several potential mechanisms: altering gene expression, the activation of oncogenes, inactivation of tumor suppressor genes, reducing genomic stability and clonal selection. Significant scientific efforts are currently dedicated to understanding the complexity of the crosstalk between the tumor and its hostile hypoxic microenvironment in order to avoid tolerance and attenuate resistance of the tumor cells.

Here we briefly reviewed the current advances in the understanding of hypoxia and its role in stemness acquisition and how tumor hypoxia and its associated pathways may interfere with CSCs plasticity that impacts their phenotype and function and how it may offer a potential target that could be exploited therapeutically. Clearly, CSC are of particular interest because they are believed to be the clonogenic core of the tumor and therefore represent the cell population that drives growth and progression. We believe that a better elucidation of the hypoxia inducing stemness can clearly help the design of more adapted anti-cancer therapies approaches. This review could help the design of innovative therapeutic treatment approaches by considering the interlink between TME and CSC plasticity and may also contribute to further understand the huge complexity of tumor plasticity in response to anti-cancer therapies. Therefore, the putative targeting of the hypoxic CSC niche would be highly effective for controlling tumor metastasis and dormant CSCs. Thus, it would be of paramount importance to identify potential actionable targets. The challenging targeting of tumor stemness in the context of TME complexity remains, however, a considerable challenge.

## Author contributions

All authors, ZR, RM, AB, KS, and CS have made substantial, direct, and intellectual contribution to the review, and approved it for publication.
